# Analysis of Serum and Urinal Copper and Zinc in Chinese Northeast Population with the Prediabetes or Diabetes with and without Complications

**DOI:** 10.1155/2013/635214

**Published:** 2013-09-22

**Authors:** Jiancheng Xu, Qi Zhou, Gilbert Liu, Yi Tan, Lu Cai

**Affiliations:** ^1^The Department of Clinical Laboratory at the First Hospital of Jilin University, Changchun 130021, China; ^2^The Department of Pediatrics at the First Hospital of Jilin University, Changchun 130021, China; ^3^The Department of Pediatrics of University of Louisville, Louisville, KY 40202, USA

## Abstract

This study investigated the association of copper and zinc levels in the serum or urine of patients living in northeast China, with either prediabetes or diabetes. From January 2010 to October 2011, patients with type 1 diabetes (T1D, *n* = 25), type 2 diabetes (T2D, *n* = 137), impaired fasting glucose (IFG, *n * = 12) or impaired glucose tolerance (IGT, *n* = 15), and age/gender matched controls (*n* = 50) were enrolled. In the T2D group, there were 24 patients with nephropathy, 34 with retinopathy, and 50 with peripheral neuropathy. Serum copper levels were significantly higher in IFG, IGT, and T2D groups. Serum zinc level was dramatically lower, and urinary zinc level was significantly higher in both T1D and T2D subjects compared with controls. The serum zinc/copper ratio was significantly lower in all the patients with IFG, ITG, T1D, and T2D. The serum copper level was positively associated with HbA1c in T2D subjects. Simvastatin treatment in T2D patients had no significant effect on serum and urinary copper and zinc. These results suggest the need for further studies of the potential impact of the imbalanced serum copper and zinc levels on metabolic syndrome, diabetes, and diabetic complications.

## 1. Introduction

Diabetes has become a pandemic disease. According to the International Diabetes Federation, diabetes affects at least 285 million people worldwide, and this number is expected to reach 438 million by the year 2030 [1]. The number of adults with impaired glucose tolerance will rise from 344 million in 2010 to an estimated 472 million by 2030 [1]. The prevalence of diabetes in China has increased dramatically in recent decades. In 1980, less than 1% of Chinese adults had this disease. By 2008, the prevalence had reached nearly 10% [1, 2]. It was estimated that more than 92 million Chinese adults had diabetes and another 148 million were prediabetic [2]. The threatening effect of diabetes for these patients is chronic hyperglycemia induced microvascular complications such as diabetic retinopathy, neuropathy, and nephropathy [2]. 

Excessive caloric intake and high-energy diet quality are major driving forces behind escalating diabetes and the appearance of epidemics worldwide [1, 3]. As an essential component of the daily diet intake, trace elements are also important for the pathogenesis of diabetes and diabetic complications. Disturbances in trace element status and increased oxidative stress in diabetes may contribute to insulin resistance and the development of diabetes and diabetic complications [4, 5]. On the other hand, progression of diabetes may also lead to perturbation in trace element metabolism and homeostasis [6]. Copper (Cu) and zinc (Zn) play critical roles in oxidant/antioxidant mechanisms. Imbalances in these processes lead to high susceptibility to oxidative damage of tissues and eventually to the development of diabetes and diabetic complications [7–12]. 

Cu is a prooxidant, participating in metal-catalyzed formation of free radicals. Cu and Zn also act as structural and catalytic components of some metalloenzymes [6]. For example, Cu is necessary for the catalytic activity of Cu/Zn superoxide dismutase (SOD) that is involved in the protection of cells from superoxide radicals [6]. Zn acts as an antioxidant by protecting the sulfhydryl groups of proteins and enzymes against free radical damage in the body [13]. Both protective and toxic effects of these metals in the pathogenesis of diabetes and diabetic complications have been documented [14–16].

Most previous studies have focused on the comparison of the serum Cu levels of diabetic subjects with nondiabetic healthy subjects. There is relatively little information about the effect of serum Cu level on the prevalence of prediabetes and diabetic patients with and without complications, focusing on Chinese populations. Therefore, we have examined Cu and Zn levels in the serum and urine of populations residing in northeast China, analyzing different subgroups of study subjects defined by insulin sensitivity and presence of diabetic complications.

## 2. Research Design and Methods

### 2.1. Ethics Statement

This study was approved by the institutional ethics committee of the First Hospital of Jilin University. Written informed consent was obtained from all subjects. For patients younger than 18 years, their parents provided written informed consent and when possible, child subjects provided written assent. 

### 2.2. Patients and Their General Information

Description of this study population has been reported in a separate publication [17] but is briefly summarized here. From January 2010 to October 2011, we enrolled 189 patients with diabetes or prediabetes plus 50 healthy control patients. The age range was 20–65 with a median age of 55 years old. The characteristics of patients by disease categories are as follows: impaired fasting glucose (IFG, *n* = 12, 8 males and 4 females, age range of 31–53), impaired glucose tolerance (IGT, *n* = 15, 9 males and 6 females with an age range of 40–56), type 1 diabetes (T1D, *n* = 25, 8 males and 17 females with an age range of 9–33 years at median age of 25 years), and T2D (*n* = 137, 85 males and 52 females, age range of 42–62 with a median age of 56 years old). The characteristics of patients with diabetes complications are as follows: patients with nephropathy (DN, *n* = 24, 19 males and 5 females, with a median age of 60 from 28 to 84 years old), patients with retinopathy (DR, *n* = 34, 15 males and 19 females, ages from 29 to 74 with a median age of 60 years old), and peripheral neuropathy (DPN, *n* = 50, 29 males and 21 females, ages from 27 to 79 with a median age of 56 years old). 

Demographic data for these patients, including age, sex, body mass index (BMI), presence or absence of diabetes, hypertension, dyslipidemia, and medication (simvastatin), were obtained from the patients' medical records. BMI was calculated as body weight (kg) divided by height (m) squared. Hypertension was defined as systolic blood pressure ≥140 mm Hg and/or diastolic blood pressure ≥90 mm Hg; IFG was defined as a fasting glucose concentration 6.1–6.9 mmol/L or nonfasting glucose concentration >7.8 mmol/L; impaired glucose tolerance (IGT) was defined as a fasting glucose <7.0 mmol/L but nonfasting glucose level in 7.8–11.0 mmol/L; diabetes was defined as a fasting glucose concentration ≥7.0 mmol/L, nonfasting glucose concentration ≥11.1 mmol/L, hemoglobin A1c (HbA1c) value ≥6.5%, and/or a previous diagnosis of diabetes; dyslipidemia was defined as a triglyceride level ≥1.7 mmol/L, total cholesterol ≥5.18 mmol/L, low density lipoprotein (LDL) ≥3.37 mmol/L, and/or previous diagnosis of dyslipidemia; estimating glomerular filtration rate (eGFR) was used as measure of kidney function.

### 2.3. Other Measurements

Laboratory data including blood glucose, HbA1c, red blood cell (RBC), hemoglobin, urea nitrogen (BUN), creatinine, total cholesterol (CHO), triglyceride (TG), high-density lipoprotein (HLDL), and low-density lipoprotein cholesterol (LDL) were measured from the first blood samples after admission. The total serum and urinal Cu and Zn, including free/active and conjugated components, were determined using inductively coupled plasma spectrometer (ICP-MS, PerkinElmer Life and Analytical Sciences, Inc., CT, USA).

Blood samples from subjects were taken after overnight fasting into commercial tubes for analysis of laboratory parameters and into special metal-free tubes for analysis of Cu and Zn. After blood centrifugation, serum was aliquoted into metal-free Eppendorf test tubes, frozen, and stored at −80°C until further analysis. 

A 24 h urine sample was obtained after admission to measure eGFR. The eGFR was calculated based on the Cockroft-Gault equation for Chinese individual: Creatinine Clearance (Ccr) = ((140 − age) × body weight)/(serum creatinine × 72) × (0.85, if female) [18].

### 2.4. Comparison of Serum Cu Levels in the T2D Patients before and after 1-Month Treatment with Simvastatin

Twenty-four patients with T2D who were not receiving any lipid-lowering drugs were recruited. Inclusion criteria were CHO > 6.22 mmol/L and LDL > 4.14 mmol/L. Exclusion criteria included receiving any lipid-lowering therapy including fish oil, probucol, vitamin E, steroid hormones, immunosuppressants, aluminum-containing antacids and erythromycin, ketoconazole or analogues, or p-aminoacetic acid. Patients were assigned to treatment for 1 month with 10 mg/day of simvastatin as clinically indicated. Blood samples were taken from fasting patients at the beginning and the end of the 1-month therapy.

### 2.5. Statistical Analysis

Continuous variables were expressed as median (interquartile range) and categorical variables as number (percent). Mann-Whitney *U* test was used for comparisons between groups, depending on the distribution. Differences in frequency of categorical variables were assessed by the chi-square test or Fisher's exact test as appropriate. Spearman rank correlation analysis was used to evaluate the correlations between serum Cu or urinary Cu level as a continuous variable and laboratory parameters. Baseline characteristics were adjusted for age, sex, BMI, hypertension, and dyslipidemia by analysis of covariance using general linear models. All reported *P* values were two-sided, and values of *P* < 0.05 were considered statistically significant. Statistical analyses were performed using SPSS 17.0.

## 3. Results

### 3.1. Study Population and Baseline Characteristics

Baseline characteristics of these subjects were summarized in Table 1. In general, we found that the age of T1D patients was significantly younger than that of control subjects and IFG, IGT, and T2D groups. The age of IFG was slightly but significantly younger than that of control and IGT and T2D subjects. Regarding gender, the percentage of males in T1D was significantly lower than that of IFG, IGT, and T2D subjects; it was not significantly different among IFG, IGT and T2D subjects. The BMI of both T1D and T2D was significantly higher than that of the control subjects. As compared to the control group, the percentages of hypertension and dyslipidemia and the levels of blood glucose were significantly higher in all IFG, IGT, T1D, and T2D subjects. HbA1c levels were higher in the IFG, T1D, and T2D groups but not significantly different than controls in the IGT group. The levels of eGFR were significantly lower in IFG group, significantly higher in both T1D and T2D groups, and not significantly different in the IGT group, compared to control subjects. 

Compared to control subjects, the serum Cu level was significantly higher in IFG, IGT, and T2D but not different in T1D (Table 1). There were no significant differences for urinary Cu levels for the IFG, ITG, T1D, and T2D groups, compared to the control group (Table 1). The serum Zn levels were dramatically lower, and the urinary Zn levels were significantly higher in both T1D and T2D subjects compared to the control, while no significant differences were observed in IFG and IGT subjects. The ratio of serum Zn to Cu has been widely used as an index of Zn relative deficiency [19, 20]. As compared to the control group, we found that the serum Zn/Cu ratio was significantly lower in all the patients with IFG, ITG, T1D, and T2D. 

There were no significant differences between groups with disease and the healthy controls for the following laboratory parameters: RBC, Hb, BUN, Cre, CHO, TG, and HDL. The serum LDL level was significantly higher in IGT and T2D subjects compared to the control group. 

### 3.2. Analysis of the Baseline Characteristics of T2D with and without Complications

Among the patients with T2D, we further compared the baseline characteristics between those who had been diagnosed with DN, DR, or DPN versus those who did not have any of these complications (Table 2). 

 We found that the age of T2D patients with one of complications including DN, DR, or DPN was significant older than that of T2D patients without diabetic complications. Except for T2D with DR for which the percentage of male was significantly lower, there was no significant gender difference in T2D patients with either DN or DPN compared to those without complication. The BMI of T2D with DN was significant higher than that of T2D subjects without complications. There was no significant difference for BMI among other patients. All T2D patients with any of these complications had significant high rates of hypertension than those without complications. There were no significant differences for the proportions of subjects with dyslipidemia or measures of blood glucose, HbA1c and Hb, between the groups defined by presence of diabetic complications. The levels of eGFR were significantly lower in the IFG group and significantly higher in T1D and T2D groups but not significantly different in the IGT group compared to controls. Regarding measures of renal function such as BUN, Cre, and eGFR, T2D patients with DN had significantly higher measures compared to T2D patients without complications. 

Serum Cu level was significantly higher only among T2D patients with DN (Table 2). The urinary Cu levels in all the T2D patients with or without complications were not significantly different (Table 2). The serum Zn level was significantly lower in T2D patients with DR, and the urinary Zn level was significantly higher in T2D patients with DPN compared to T2D patients without complications. The ratio of serum Zn/Cu was significantly lower in T2D patients with DN and DR compared to T2D patients without complications. No significant differences in serum levels of CHO, TG and LDL were observed in any T2D groups. Serum HDL levels were significantly higher in T2D patients with DN. 

### 3.3. Associations of Serum or Urinary Cu Levels with Laboratory Parameters in Subjects

We analyzed associations of the serum or urinary Cu level with other laboratory parameters in all subjects (Table 3). In control subjects, serum Zn levels were positively correlated with serum Cu. In IFG patients, urinary Cu levels were positively associated with urinary Zn levels. In IGT patients, serum Cu levels were positively associated with serum HDL. 

In T1D patients, serum Cu levels were negatively associated with blood glucose levels and positively associated with serum Zn levels. Urinary Cu levels were negatively associated with serum CHO, HDL, and LDL levels.

For T2D patients, serum Cu levels were positively associated with serum HbA1c, serum BUN, and urinary Cu; urinary Cu levels were positively associated with urinary Zn and serum BUN and Cre levels but negatively associated with eGFR levels. 

### 3.4. Association of Serum or Urinary Cu Levels with Laboratory Parameters in T2D with or without Complications

**Figure 1:**
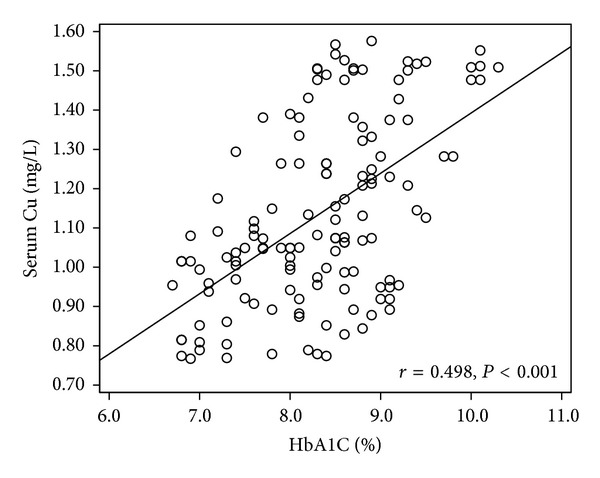
Correlations between Cu with HbA1c in T2D patients. Correlations between serum Cu and HbA1c in T2D patients (*r* = 0.498, *P* < 0.001).

We further analyzed the association of the serum or urinary Cu levels with the laboratory parameters in T2D subjects with or without complications (Table 4). Serum Cu levels were positively associated with HbA1c levels in all the T2D patients with and without complications (Figure 1). In contrast, serum Cu levels were positively associated with blood glucose levels only in T2D patients with DPN (Table 4).

Serum Zn was negatively correlated with urinary Cu in patients with DN. Urinary Zn was negatively correlated with serum Cu in patients with DN and positively correlated with both serum and urinary Cu in patients with DPN. 

Serum Cu levels were negatively associated with serum LDL in T2D patients without complications. Urinary Cu levels were positively associated with serum Cre in patients with DPN and also with serum HDL in the DN group but negatively associated with eGFR and serum Cu level in T2D patients without complications. 

### 3.5. Effect of Simvastatin Treatment on Cu Levels and Other Variables in T2D Patients

Since T2D patients were often treated with statins to lower their lipid profiles, we examined the effect of 1-month simvastatin treatment on serum and urinary Zn and Cu in T2D patients (Table 5). Treatment with simvastatin for 1 month significantly reduced CHO and LDL levels as expected [17]. One month of simvastatin therapy had no significant effects on the levels of serum and urinary Zn, Cu and Zn/Cu among T2D patients.

## 4. Discussion

### 4.1. Serum Cu and Zn in Prediabetes, and Diabetes

The association of serum Cu and Zn levels with diabetes has been extensively studied [21–25]. These previous studies have demonstrated that serum Cu levels are significantly increased, and Zn levels are significantly decreased in adult T2D [21–23, 25]. Among studies of younger adults (age = 27 ± 6.2) with T2D [24], both Cu and Zn levels are significantly decreased. To our knowledge, this is the first study to report analyses of Cu and Zn among patients with diabetes or prediabetes for populations residing in northeast China. We directly measured serum concentrations of Cu by means of an atomic absorption spectrophotometer and identified some inconsistency with published studies. We observed that serum concentration of Cu is significantly high and Zn is significantly low in T2D compared to the control subjects, which are consistent with previous studies in adult T2D patients [21–23]. The literature regarding serum Cu level in patients with T1D is very limited. In our study, we found that serum Cu levels in patients with T1D are not significantly different from matched control subjects without diabetes, but serum Zn is significantly (*P* = 0.056) reduced compared to the control subjects. This finding contrasts with an earlier report [26], in which they found that the concentrations of serum Cu in patients with T2D are significantly higher than control subjects. This may suggest that there are different profiles among populations residing in different countries, perhaps related to substantial variation in dietary composition according to different geographic territory. The changes of serum Zn levels in T1D have been inconsistently documented [26–30]. Some of the studies found that the serum Zn levels are significantly decreased [26, 28], while others studies report no significant differences [27]. 

 In the present study, we demonstrated for the first time that prediabetic subjects including both IFG and IGT subjects exhibited significantly increased serum Cu levels but no significant change for serum Zn levels, compared to matched control subjects. These results imply that the increased serum Cu may increase oxidative stress and subsequent inflammation, leading to the insulin resistance and eventual development of diabetes [4, 5].

The urinary Cu levels were not significantly changed in either patients with IFG and IGT or in patients who had progressed to T1D or T2D. Urinary Zn levels were significantly increased only in T1D and T2D patients, which indicate that the low level of serum Zn in T2D may attribute to the high urinary excretion. 

It should be noted that the ratio of serum Zn to Cu, as an index of Zn relative deficiency, has been found to be significantly decreased in type 1 diabetic patients [6]; this was corroborated in the present study for IFG, IGT, T2D, and T1D patients. This suggests that the ratio of serum Zn level to Cu level may be a more accurate predictor than absolute levels of serum Zn or Cu. Looking in isolation at either Zn or Cu may result in inaccuracy since individually assessing either element is vulnerable to increased variation stemming from individual differences related to dietary intake. 

### 4.2. Serum or Urinary Cu and Diabetic Complications

Several studies have reported significant associations between serum and/or urinary Cu levels and the risk of various diabetic complications [6, 24, 31–33]. Except for higher levels of serum Cu observed in subjects with DN, we did not find any significant difference for either serum or urinary Cu among uncomplicated T2D patients or T2D patients with either DR or DPN. An earlier study reported no significant differences for either Zn or Cu levels, as well as the Zn/Cu ratio in diabetic patients with and without complications [34]. Another cross-sectional study that evaluated 24 h urinary Cu levels in T2D patients (*n* = 42) with DN in comparison with patients without DN (*n* = 40) reported that diabetic patients with DN have increased urinary Cu excretion [31]. 

These contrasting findings among studies comparing serum or urinary Cu levels among patients with diabetic complications may depend on the stage of pathogenesis for diabetes morbidity. It is known that increased Cu may increase prooxidant stress and weaken antioxidant defense, resulting in progressive damage to the blood vessels, heart, kidneys, retina, and nerves [35, 36]. Accumulating evidence from diabetic patients and animal models demonstrates that treatment with Cu specific chelator can significantly prevent and reduce diabetes-induced cardiac and renal complications [36–40], strongly supporting the important pathogenic role of increased systemic Cu in the development of diabetic complications.

One possible mechanism by which increased systemic Cu levels can harm diabetic patients may be related to its effect on hemoglobin glycation, shown by the increased level of HbA1c in T2D. Numerous studies have indicated that minimizing hyperglycemia is an effective approach to prevent diabetic complications [41–44]. The association between systemic Cu levels and hyperglycemia control situation has been rarely documented. We demonstrated a significantly positive association between serum Cu with serum HbA1c in T2D patients (Table 4 and Figure 1). This result is consistent with a newly published study that examines the association of serum Cu levels and glycemic control in 132 patients with T2D [21]. They found that serum Cu levels were positively correlated with HbA1c levels. In addition, after 3-month glycemic control, they further demonstrated that as hemoglobin HbA1c levels decreased (from 8.7% to 6.8%, *P* < 0.001), Cu levels tended to decrease (from 105.7 mug/dL to 101.8 mug/dL, *P* = 0.069). Whether serum Cu levels can be used as a marker to distinguish T2D with complications from uncomplicated T2D patients remains unclear. We did not observe a significant difference in HbA1c when comparing patients diagnosed with T2D complications compared to uncomplicated T2D patients (Table 2). We did observe that serum Cu levels were significantly associated with HbA1c levels in uncomplicated T2D patients (Table 4, *P* = 0.041) and that serum Cu levels were significantly associated with HbA1c in patients with DN (*P* = 0.032), DR (*P* = 0.017), and DPN (*P* < 0.001). Further clinical investigation with larger numbers of cases from different geographic regions is needed.

### 4.3. Effect of Statin Treatment on Serum or Urinary Cu

There are few reports regarding the effect of statin therapy on the levels of serum or urinary Cu in patients with diabetes. We found no significant differences in serum or urinary Cu and Zn in the T2D patients before and after treatment with simvastatin (Table 5). Statins are widely used in the management of coronary risk because of their efficacy in reducing LDL and their potentially protective pleiotropic effects. A study that investigated the effect of simvastatin on serum Zn, Cu, and Zn/Cu ratio with other measurements in dyslipidemic patients (*n* = 72) did not identify any significant associations for the serum levels of Zn, Cu, and Zn/Cu ratio before and after simvastatin treatment [45]. Another study, in which patients previously not treated with a lipid-lowering agent (*n* = 20) were treated with either simvastatin (*n* = 11) or atorvastatin (*n* = 9) for 4 months, showed that statin treatment was associated with a significant reduction in mean serum Zn and Cu. The effect of statin treatment on these serum trace elements may be influenced by the statin type as well as treatment dose and duration.

In summary, the present study investigated the association of serum and urinary Cu and Zn levels for northeast Chinese patients with either prediabetes or diabetes with and without complications. Compared to previously published data from other geographic regions, these diabetic patients predominantly exhibited similar profiles for these trace elements but did demonstrate a few inconsistent patterns. We clearly showed the positive association of serum Cu levels with Hb1Ac levels in all T2D patients; however, whether the effect of serum Cu on Hb1Ac is related to the progression of diabetes and the development of diabetic complications remains unclear and in need of further exploration.

## Figures and Tables

**Table 1:** Baseline characteristics of subjects.

	CON (*n* = 50)	IFG (*n* = 12)	*P*	IGT (*n* = 15)	*P*	T1D (*n* = 25)	*P*	T2D (*n* = 137)	*P*
Age (years)	55.0 (45.0–63.0)	50.5 (43.0–51.8)	0.038*	53.0 (49.0–55.0)	0.285	25.0 (19.0–30.5)	<0.001*	56.0 (46.0–63.0)	0.521
Sex, male (%)	31 (62.0)	8 (66.7)	0.764	9 (60.0)	0.889	8 (32.0)	0.014*	85 (62.0)	0.996
BMI (kg/m^2^)	21.6 (19.5–22.9)	22.4 (20.5–27.6)	0.110	22.1 (20.4–24.0)	0.055	22.9 (20.3–27.5)	0.002*	25.4 (23.3–27.6)	<0.001*
Hypertension (%)	0	3 (25.0)	<0.001*	4 (26.7)	<0.001*	15 (60.0)	<0.001*	58 (42.3)	<0.001*
Dyslipidemia (%)	0	4 (33.3)	<0.001*	5 (33.3)	<0.001*	22 (88.0)	<0.001*	77 (56.2)	<0.001*
Glu (mmol/L)	4.8 (4.1–5.6)	6.4 (6.3–6.7)	<0.001*	5.9 (5.6–6.1)	0.019*	11.9 (8.5–14.8)	<0.001*	8.8 (7.5–11.5)	<0.001*
HbA1c (%)	5.1 (4.2–6.0)	6.1 (5.9–6.2)	0.017*	5.5 (5.4–5.7)	0.278	11.8 (10.3–14.2)	<0.001*	8.4 (7.7–8.9)	<0.001*
RBC (×10^12^)	4.8 (4.3–5.0)	4.9 (4.3–5.0)	0.830	4.8 (4.4–5.0)	0.604	5.0 (4.6–5.2)	0.229	4.7 (4.3–5.1)	0.344
Hb (g/L)	133.2 (128.1–153.7)	143.5 (131.3–148.0)	0.837	142.0 (132.0–148.0)	0.578	146.0 (135.0–155.0)	0.324	141.0 (129.0–154.0)	0.791
BUN (mmol/L)	5.5 (4.2–6.4)	6.5 (5.4–7.2)	0.389	4.5 (3.6–5.4)	0.400	5.7 (4.3–6.6)	0.063	6.2 (5.2–8.3)	0.938
Cre (*μ*mol/L)	73.9 (63.1–80.9)	66.7 (60.3–70.2)	0.951	64.5 (56.9–74.1)	0.138	71.5 (61.6–88.1)	0.286	77.5 (64.6–113.6)	0.657
eGFR (mL/min)	24.6 (22.7–26.4)	21.9 (20.2–25.2)	0.009*	25.4 (22.8–27.0)	0.487	115.7 (83.8–143.5)	<0.001*	87.4 (54.0–112.7)	<0.001*
Cu (mg/L)	0.80 (0.70–0.99)	1.13 (1.02–1.29)	0.001*	1.15 (1.03–1.24)	<0.001*	0.94 (0.75–1.19)	0.352	1.07 (0.95–1.33)	<0.001*
UCu (*μ*g/L)	25.0 (22.0–40.0)	32.0 (28.3–42.5)	0.293	26.0 (24.0–28.0)	0.306	25.0 (23.0–27.5)	0.187	30.0 (25.5–40.5)	0.408
Zn (mg/L)	0.81 (0.67–0.93)	0.75 (0.70–0.84)	0.462	0.77 (0.67–0.87)	0.834	0.59 (0.53–0.75)	0.056	0.61 (0.51–0.75)	<0.001*
UZn (mg/L)	0.20 (0.14–0.32)	0.32 (0.26–0.37)	0.745	0.27 (0.19–0.41)	0.836	0.86 (0.67–0.91)	<0.001*	0.48 (0.38–0.57)	<0.001*
Zn/Cu	0.92 (0.84–1.07)	0.68 (0.62–0.73)	0.009*	0.65 (0.62–0.77)	0.002*	0.64 (0.57–0.77)	0.018*	0.56 (0.43–0.68)	<0.001*
CHO (mmol/L)	4.5 (3.2–5.1)	4.8 (4.5–5.1)	0.228	4.7 (4.2–5.0)	0.169	5.4 (4.4–5.7)	0.548	5.0 (4.4–6.0)	0.594
TG (mmol/L)	1.2 (1.0–1.4)	1.4 (1.3–1.6)	0.893	1.3 (1.2–1.4)	0.079	1.8 (1.1–2.8)	0.346	1.6 (1.1–2.7)	0.490
HDL (mmol/L)	1.2 (1.1–1.3)	1.1 (0.9–1.2)	0.981	1.1 (1.0–1.2)	0.619	1.1 (1.0–1.5)	0.095	1.2 (1.0–1.5)	0.665
LDL (mmol/L)	2.4 (2.0–2.9)	3.0 (2.5–3.2)	0.464	2.9 (2.6–3.1)	0.002*	3.5 (2.8–4.2)	0.763	3.4 (2.7–3.8)	0.044*

Data are presented as number (%) or median (interquartile range). Baseline characteristics were adjusted for age, sex, BMI, hypertension and dyslipidemia by analysis of covariance using general linear models. *P < 0.05 versus control group. BMI: body mass index, Glu: serum glucose, HbA1c: glycated hemoglobin, RBC: red blood cell, Hb: hemoglobin, BUN: blood urea nitrogen, Cre: serum creatinine, eGFR: estimating GFR, Cu: serum copper, UCu: urinary copper, Zn: serum zinc, UZn: urinary zinc, CHO: total cholesterol, TG: triglyceride, HDL: high-density lipoprotein cholesterol, LDL: low-density lipoprotein cholesterol.

**Table 2:** Baseline characteristics of T2D subjects.

	T2D Con (*n* = 29)	DN (*n* = 24)	*P*	DR (*n* = 34)	*P*	DPN (*n* = 50)	*P*
Age (years)	46.0 (39.0–60.0)	60.0 (46.8–65.0)	0.007*	59.5 (46.8–67.3)	0.003*	55.5 (46.8–62.3)	0.007*
Sex, male (%)	22 (75.9)	19 (79.2)	0.775	15 (44.1)	0.011*	29 (58.0)	0.110
BMI (kg/m^2^)	25.4 (23.0–27.3)	27.1 (24.9–30.4)	0.026*	24.8 (22.4–27.4)	0.539	25.1 (23.0–26.5)	0.618
Hypertension (%)	0	21 (87.5)	<0.001*	17 (50.0)	<0.001*	20 (40.0)	<0.001*
Dyslipidemia (%)	20 (69.0)	11 (45.8)	0.089	16 (47.1)	0.080	27 (54.0)	0.192
Glu (mmol/L)	9.8 (8.3–12.8)	8.2 (7.5–9.5)	0.248	9.2 (7.7–11.4)	0.182	8.8 (7.6–11.1)	0.413
HbA1c (%)	8.5 (7.7–8.9)	8.5 (8.0–8.8)	0.380	8.6 (8.0–8.9)	0.849	8.2 (7.2–8.8)	0.421
RBC (×10^12^)	5.1 (4.6–5.4)	4.8 (4.0–5.1)	0.224	4.6 (4.2–4.9)	0.395	4.7 (4.3–4.9)	0.027*
Hb (g/L)	154.0 (137.0–161.5)	144 (129.8–159.5)	0.076	138.0 (125.8–151.0)	0.760	140.0 (128.5–150.0)	0.067
BUN (mmol/L)	5.6 (4.7–6.5)	17.5 (13.2–19.1)	<0.001*	5.9 (5.2–7.4)	0.430	5.9 (4.2–6.9)	0.525
Cre (*μ*mol/L)	70.2 (58.6–85.6)	287.0 (233.9–386.3)	<0.001*	79.0 (65.0–107.0)	0.095	71.0 (61.4–87.6)	0.824
eGFR (mL/min)	102.8 (87.4–166.2)	25.7 (16.8–34.9)	<0.001*	85.5 (58.2–120.0)	0.182	98.0 (72.8–112.6)	0.530
Cu (mg/L)	1.00 (0.94–1.15)	1.26 (1.07–1.42)	0.015*	1.05 (0.92–1.48)	0.717	1.08 (0.92–1.38)	0.298
UCu (*μ*g/L)	29.0 (24.5–36.5)	37.0 (28.3–49.0)	0.947	28.5 (26.8–36.0)	0.274	32.0 (22.8–43.0)	0.815
Zn (mg/L)	0.73 (0.55–0.79)	0.59 (0.48–0.76)	0.157	0.58 (0.46–0.63)	0.002*	0.63 (0.59–0.75)	0.080
UZn (mg/L)	0.47 (0.28–0.53)	0.44 (0.30–0.52)	0.685	0.45 (0.25–0.52)	0.824	0.52 (0.44–0.63)	<0.001*
Zn/Cu	0.66 (0.55–0.76)	0.50 (0.36–0.56)	0.006*	0.48 (0.40–0.64)	0.012*	0.58 (0.46–0.68)	0.111
CHO (mmol/L)	5.0 (4.4–5.5)	5.7 (4.6–6.5)	0.858	4.9 (4.5–6.3)	0.379	4.9 (4.3–6.0)	0.961
TG (mmol/L)	1.7 (1.1–2.5)	2.0 (1.2–3.3)	0.457	1.4 (1.1–2.8)	0.734	1.3 (1.0–2.6)	0.726
HDL (mmol/L)	1.2 (0.9–1.9)	1.1 (1.0–1.5)	0.014*	1.2 (1.1–1.6)	0.616	1.1 (1.1–1.4)	0.700
LDL (mmol/L)	3.3 (2.4–3.7)	3.5 (2.9–4.1)	0.919	3.4 (2.7–3.8)	0.335	3.3 (2.6–3.9)	0.703

Data presentation and abbreviations spelt out forms are the same as the description for Table 1. *P < 0.05 versus T2D control group.

**Table 3:** Associations between serum Cu or urinary Cu level as a continuous variable and laboratory parameters in subjects.

	CON (*n* = 50)				IFG (*n* = 12)				IGT (*n* = 15)				T1D (*n* = 25)				T2D (*n* = 137)			
	Cu		UCu		Cu		UCu		Cu		UCu		Cu		UCu		Cu		UCu	
	*r*	*P*	*r*	*P*	*r*	*P*	*r*	*P*	*r*	*P*	*r*	*P*	*r*	*P*	*r*	*P*	*r*	*P*	*r*	*P*
Glu	−0.157	0.214	−0.186	0.196	−0.004	0.991	−0.071	0.826	0.011	0.970	−0.047	0.867	−0.530	0.006*	0.225	0.279	0.109	0.206	0.018	0.838
HbA1c	−0.060	0.681	−0.109	0.451	0.569	0.054	−0.066	0.838	0.135	0.631	0.120	0.671	−0.035	0.867	−0.109	0.603	0.498	<0.001*	0.046	0.591
RBC	0.125	0.389	−0.231	0.106	0.241	0.450	−0.240	0.452	−0.054	0.849	0.092	0.746	0.059	0.781	0.136	0.516	−0.098	0.255	0.073	0.395
Hb	−0.007	0.959	−0.151	0.295	0.308	0.330	−0.131	0.685	−0.011	0.970	0.085	0.763	0.228	0.273	0.256	0.216	−0.036	0.673	0.039	0.650
UCu	−0.204	0.154	—	—	0.237	0.459	—	—	0.284	0.305	—	—	0.096	0.649	—	—	0.184	0.032*	—	—
Zn	0.298	0.036*	−0.121	0.402	0.116	0.720	0.057	0.861	0.118	0.676	−0.186	0.506	0.403	0.046*	0.140	0.506	0.158	0.065	−0.048	0.581
UZn	−0.017	0.906	−0.043	0.767	0.193	0.543	0.673	0.017*	0.118	0.676	0.063	0.823	0.060	0.775	0.202	0.333	0.048	0.577	0.204	0.017*
BUN	0.025	0.866	−0.059	0.685	−0.179	0.579	0.189	0.556	−0.203	0.469	0.113	0.690	−0.186	0.374	0.130	0.536	0.248	0.001*	0.248	0.003*
Cre	−0.090	0.533	0.040	0.781	−0.427	0.166	−0.347	0.269	0.143	0.611	0.302	0.273	0.092	0.663	0.329	0.109	0.125	0.145	0.357	<0.001*
eGFR	0.108	0.457	−0.003	0.983	0.329	0.296	0.366	0.241	0.077	0.785	−0.332	0.226	−0.025	0.904	−0.034	0.873	−0.146	0.090	−0.337	<0.001*
CHO	0.044	0.762	0.152	0.292	0.193	0.548	0.179	0.578	−0.349	0.202	−0.221	0.428	0.198	0.342	−0.490	0.013*	0.119	0.165	0.032	0.708
TG	0.092	0.526	0.151	0.295	0.199	0.536	0.522	0.082	−0.116	0.681	−0.270	0.330	−0.023	0.911	0.268	0.196	0.076	0.379	−0.046	0.597
HDL	−0.148	0.307	−0.053	0.717	−0.046	0.887	−0.159	0.620	0.556	0.032	0.015	0.958	−0.151	0.472	−0.476	0.016*	0.132	0.124	−0.017	0.845
LDL	−0.007	0.963	−0.032	0.827	0.359	0.252	0.103	0.750	0.120	0.670	0.180	0.520	0.101	0.630	−0.540	0.005*	0.128	0.137	0.151	0.079

Data presentation and abbreviations spelt out forms are the same as the description for Table 1. *P < 0.05 for the association.

**Table 4:** Associations between serum Cu or urinary Cu level as a continuous variable and laboratory parameters in T2D subjects.

	T2D Con (*n* = 29)				DN (*n* = 24)				DR (*n* = 34)				DPN (*n* = 50)			
	Cu		UCu		Cu		UCu		Cu		UCu		Cu		UCu	
	*r*	*P*	*r*	*P*	*r*	*P*	*r*	*P*	*r*	*P*	*r*	*P*	*r*	*P*	*r*	*P*
Glu	0.095	0.624	−0.180	0.351	0.289	0.171	0.321	0.127	0.016	0.929	0.129	0.468	0.296	0.037*	0.046	0.753
HbA1c	0.382	0.041*	−0.307	0.106	0.440	0.032*	0.194	0.365	0.406	0.017*	0.031	0.863	0.630	<0.001*	0.161	0.264
RBC	−0.138	0.477	−0.080	0.679	−0.136	0.525	0.134	0.532	−0.039	0.828	−0.093	0.601	0.017	0.906	0.127	0.381
Hb	0.093	0.63	−0.133	0.491	−0.145	0.500	0.084	0.697	0.125	0.483	−0.006	0.971	−0.031	0.833	0.073	0.612
UCu	−0.210	0.273	—	—	−0.067	0.755	—	—	0.116	0.514	—	—	0.258	0.071	—	—
Zn	0.342	0.069	0.117	0.168	0.281	0.184	−0.493	0.014*	0.105	0.553	−0.195	0.270	0.219	0.127	0.136	0.347
UZn	0.224	0.243	0.545	0.382	−0.481	0.017*	0.179	0.403	−0.048	0.786	0.028	0.873	0.327	0.021*	0.319	0.024*
BUN	0.042	0.828	0.350	0.062	0.087	0.687	0.152	0.479	0.033	0.854	−0.146	0.411	0.200	0.164	0.148	0.305
Cre	−0.322	0.089	0.283	0.137	−0.164	0.445	0.053	0.807	0.020	0.912	0.261	0.137	−0.099	0.492	0.320	0.023*
eGFR	0.083	0.670	−0.372	0.047*	0.040	0.852	−0.115	0.591	0.086	0.630	−0.109	0.539	0.068	0.641	−0.276	0.052
CHO	−0.321	0.090	0.075	0.699	0.154	0.472	−0.117	0.586	0.010	0.955	−0.173	0.329	0.192	0.181	0.077	0.595
TG	−0.152	0.433	0.061	0.752	0.080	0.711	−0.278	0.189	0.016	0.930	−0.092	0.607	0.148	0.307	−0.058	0.689
HDL	0.147	0.446	−0.049	0.799	−0.090	0.675	0.420	0.041*	0.189	0.286	−0.273	0.118	0.107	0.459	−0.038	0.795
LDL	−0.397	0.033*	0.049	0.800	0.198	0.353	0.197	0.357	0.132	0.457	0.003	0.986	0.198	0.168	0.156	0.281

Data presentation and abbreviations spelt out forms are the same as the description for Table 1. *P < 0.05 for the association.

**Table 5:** Serum parameters in T2D patients treated with simvastatin.

	Simvastatin (*n* = 24)		
	Pretreatment	Posttreatment	*P*
Cu (mg/L)	1.18 (0.94–1.62)	1.11 (0.99–1.64)	0.765
UCu (*μ*g/L)	24.5 (19.0–31.5)	21.5 (17.3–31.0)	0.445
Zn (mg/L)	0.65 (0.47–0.94)	0.53 (0.43–0.77)	0.108
UZn (mg/L)	0.36 (0.17–0.57)	0.25 (0.13–0.42)	0.097
Zn/Cu	0.58 (0.34–0.96)	0.48 (0.39–0.68)	0.592

Data presentation and abbreviations spelt out forms are the same as the description for Table 1. *P < 0.05 versus pretreatment.
